# An unusual 100-million-year old holometabolan larva with a piercing mouth cone

**DOI:** 10.7717/peerj.8661

**Published:** 2020-04-03

**Authors:** Joachim T. Haug, Mario Schädel, Viktor A. Baranov, Carolin Haug

**Affiliations:** 1 Department of Biology II, Ludwig-Maximilians-Universität München, Planegg-Martinsried, Germany; 2 GeoBio-Center at LMU, München, Germany

**Keywords:** Holometabola, Insecta, Evolution, Mouth parts, Convergence

## Abstract

Holometabola is a hyperdiverse group characterised by a strong morphological differentiation between early post-embryonic stages (= larvae) and adults. Adult forms of Holometabola, such as wasps, bees, beetles, butterflies, mosquitoes or flies, are strongly differentiated concerning their mouth parts. The larvae most often seem to retain rather plesiomorphic-appearing cutting-grinding mouth parts. Here we report a new unusual larva preserved in Burmese amber. Its mouth parts appear beak-like, forming a distinct piercing mouth cone. Such a morphology is extremely rare among larval forms, restricted to those of some beetles and lacewings. The mouth parts of the new fossil are forward oriented (prognathous). Additionally, the larva has distinct subdivisions of tergites and sternites into several sclerites. Also, the abdomen segments bear prominent protrusions. We discuss this unusual combination of characters in comparison to the many different types of holometabolan larvae. The here reported larva is a new addition to the ‘unusual zoo’ of the Cretaceous fauna including numerous, very unusual appearing forms that have gone extinct at the Cretaceous–Palaeogene boundary.

## Introduction

The myriad representatives of Insecta (Hexapoda in Anglo-American tradition) are very successful in evolutionary terms concerning species richness, biomass, number of individuals, number of ecological roles or whatever measure we suggest. This success has been attributed to numerous different factors—two will be considered with some more detail here: (1) the very diverse modifications of their feeding apparatus (Grimaldi & Engel, 2005; Engel, 2018) and (2) the differentiation of the resources exploited by larval and adult representatives of Holometabola (Grimaldi & Engel, 2005), a major and especially species-rich ingroup of Insecta (including beetles, wasps, butterflies and so on; Grimaldi & Engel, 2005).

Larvae and adults of Holometabola are remarkably diverse in their morphology (Engel, 2018). The range of morphological diversity varies from the larvae of Megaloptera where both adults and larvae have fully functional jointed legs and quite similar mouth parts, to larvae of the dipteran ingroup Cyclorrhapha, where headless and legless larvae (maggots) are in strong contrast to nimble winged and large-eyed adults (flies) (Engel, 2018). Many representatives of Neuropteriformia, especially of Neuroptera and Coleoptera, also seem to have a huge disparity between the adults and larvae, as well as an exceedingly high diversity of the larval forms (Badano et al., 2018; Haug, Müller & Haug, 2019). Additionally, lacewings (representatives of Neuroptera) in particular seem to have a large variety of quite different larval morphotypes, many of which have not survived into the modern times (Haug, Müller & Haug, 2019).

The representatives of the different lineages of Insecta are known to possess quite different types of feeding strategies (Engel, 2018) and, of course, coupled to this, rather different morphologies of their feeding apparatuses. Interestingly, we can recognise quite a different evolutionary pattern within Insecta if we compare them to their closer relatives. Those relatives are different groups of crustaceans, as Insecta is nothing less than a group of crustaceans that includes a lineage with flying forms. Different groups of crustaceans tend to differ in the number of structures included into the feeding process. All modern crustaceans, thus also representatives of Insecta, incorporate the labrum, the mandibles, the maxillulae (maxillae in Insecta) and the maxillae (labium in insectan terminology) into the feeding apparatus, although the latter might resemble further posterior appendages in some eucrustacean ingroups (Haug et al., 2013a). Additionally, representatives of Decapoda (prawns, shrimps, lobsters, crabs and alike) add three pairs of transformed thorax appendages, so-called maxillipeds, into the feeding apparatus (Kropp, 1986). Most representatives of Isopoda (for example woodlice) have one pair of maxillipeds (Green, 1957; Brusca, 1981; Al-Zahaby, El-Aal & El-Bar, 2001), representatives of Stomatopoda (mantis shrimps) have five pairs of maxillipeds (Haug et al., 2012) and many small-sized crustaceans, such as brine shrimps have all their appendages involved in feeding (Criel & Macrae, 2002; Hegna & Lazo-Wasem, 2010).

Representatives of Insecta do (usually) not include further structures into their feeding apparatus. They simply modify the already existing structures (Snodgrass, 1993). Although representatives of some groups, for example mantodeans, use the anterior trunk appendages for catching prey and are hence involved in the feeding process, still these appendages do not become additional mouth parts as, for example, in decapodan crustaceans (Hörnig, Haug & Haug, 2017).

In some groups of Insecta the former cutting-grinding mandibles become more fixed and elongate, needle-like and together with similarly specialised mouth parts, form distinct mouth cones or piercing stylets (Beutel et al., 2013). While also some other crustaceans form piercing-sucking mouth cones (mostly parasitic forms such as representatives of Ascothoracida, Branchiura and some forms of Isopoda), those of, for example, adult mosquitoes or true bugs (Heteroptera) appear unique among crustaceans concerning their degree of modification (Wenk, Lucic & Betz, 2010, but see Nagler & Haug (2016)). Also, many other forms besides piercing and cutting type mouth parts have evolved, such as the siphoning mouth parts in bees, or sucking (but not piercing) mouth parts in derived butterflies (Krenn & Penz, 1998; Krenn, Zulka & Gatschnegg, 2001; Krenn, Plant & Szucsich, 2005; Krenn, 2010).

Notably, it seems that the most extreme modifications of mouth parts are either known from adults, or from immatures resembling the adults with respect to the mouth parts and other morphological structures (Peñalver & Pérez-De La Fuente, 2014; Baranov et al., 2019), that is, immatures of non-holometabolous insects, also called nymphs in Anglo-American literature (Bybee et al., 2015; Redei & Štys, 2016; Sahlen et al., 2016). A common differentiation of immature (mostly larval; see Haug, 2020 for the discussion) and adult mouth parts in various crustacean lineages involves a post-embryonic reduction of the distal parts, especially in the mandible (Haug & Haug, 2015). This is related to the mandible morphology in Insecta; already in the ground pattern of Insecta (≈ character set of the direct ancestor) the distal region of the mandibles appears to have been reduced, also in early stages, as evident from the lack of the *dll* patterning gene expression in the mandibles (Gotoh et al., 2017). This gene is normally taking part in the longitudinal patterning of the distalmost parts of the appendages of representatives of Euarthropoda (Gotoh et al., 2014, 2017).

Larvae seem to retain plesiomorphic (= ancestral) appearing mouth parts more often than non-holometabolan immatures or adults. Yet, this is of course an oversimplification. The immatures of odonatans (damselflies and dragonflies), often termed naiads (but ‘Larve’ in German literature, see e.g. Haug, 2020), have highly specialised mouth parts with a complex folding, thrusting and grasping mechanism in the labium. Here, adults are likely retaining a more plesiomorphic morphology (Merritt & Cummins, 1996; Lancaster & Downes, 2013). While it may sometimes not seem obvious, mouth parts of larvae of Holometabola are the same structures as in their adults and not separate entities, from a developmental perspective (Gotoh et al., 2014, 2017).

Comparably, the larvae of lacewings have highly modified mouth parts: the lower (posterior) side of the elongated mandible possesses a special groove, which is closed to a functional tube by a likewise elongated maxilla (Aspöck, Plant & Nemeschkal, 2001; Aspöck, Haring & Aspöck, 2012; Beutel, Friedrich & Aspöck, 2010; Jandausch et al., 2018). Hence, lacewing larvae possess a pair of piercing-sucking stylets, formed by interconnected mouth parts. Also some larval forms of different beetle groups have piercing mouthparts (Costa, Ide & Teixeira, 1995; Balke, 2005; Faucheux, 2014).

Still, in most groups of Holometabola highly modified mouth parts seem restricted to the adult form (Beutel et al., 2013). The larval forms often retain cutting-grinding mandibles and associated mouth parts.

In this study we present a fossil holometabolan larva with a very unusual combination of characters, including piercing mouth parts, from early Late Cretaceous Burmese amber (about 100 million years old). The morphology of the fossil larva is compared to morphologies in various holometabolan groups and possible phylogenetic relationships are discussed.

## Materials and Methods

### Material

A single piece of amber (= burmite) comes from the about 100-million-year old Burmese deposits, Hukawng Valley, Kachin State, Myanmar (Cruickshank & Ko, 2003). It was bought by Patrick Müller (Käshofen), was part of his private collection under the repository number BUB 2787, and is now deposited in the Staatliche Naturwissenschaftliche Sammlungen Bayerns–Bayerische Staatssammlung für Paläontologie und Geologie in Munich with the collection number SNSB—BSPG 2019 I 171.

The original amber piece was first cut with a Dremel 3000 Variable Speed Rotary Tool. Afterwards it was polished with wet sandpaper, first grade 200 and then subsequently grades 600, 1000 and 5000. Final polishing was performed with Sidol metal polish (Haug, Müller & Haug, 2018).

Comparative extant material came from the entomological collection of the Centrum für Naturkunde (CeNak), Hamburg. Specimens are registered under the repository numbers ZMH 62827, 62844, 62854 and 62686.

### Documentation and image processing

The specimen has been documented with different imaging techniques. In all cases distilled water was used as immersion liquid and a cover slip was placed on top of the amber piece to create an even surface, further reducing distortions (Haug, Müller & Sombke, 2013).

Overview images were recorded with a Keyence VHX-6000 digital microscope, either with ring light illumination or cross-polarised co-axial illumination (Haug, Müller & Haug, 2018). All images are composite images; each image detail was documented with a stack of images (frames) of shifting focal planes which were fused to a single sharp image with the built-in software. Several adjacent image details were stitched to a large panorama image with the built-in software, resulting in a high-resolution image. Additionally, the HDR function was employed (Haug et al., 2013b), that is, each single frame is a composite from several images under different exposure times; the resulting image contains all information without too dark or too bright regions in the image.

The head region was additionally documented on a Keyence BZ-9000 fluorescence microscope under green light (TRITC filter, 543 nm) with a 10x lens and 2x optical zoom resulting in a magnification of approximately 200×. Other body parts were also documented on the same microscope but with translucent light. Also here stacks of images were recorded. The resulting images were fused in CombineZP (Alan Hadley, GPL license) (Haug, Haug & Ehrlich, 2008; Haug et al., 2009a, 2011a, 2011b). Due to difficulties with the fusion algorithms with high magnification microscopic images in amber, in some cases only smaller sub-stacks of images were fused and the sharp areas were combined to a single image using GIMP (GPL license).

Additionally, the entire specimen was documented under phase contrast transmitted light settings with a 20x lens on the BZ-9000 fluorescence microscope. Stacks were recorded and processed in ImageJ with find edges mode and z-stack projection (maximum intensity); resulting images were stitched in Adobe Photoshop Elements 11 (modified from Haug et al. (2009b)). Noisy background was removed manually in Adobe Photoshop CS2. Colour-coded images were also prepared in Adobe Photoshop CS2.

Comparative specimens were documented under Leica dissection microscopes either with a Leica camera or a DCM 510 ocular camera. Images were processed with CombineZM (fusion of stacks) and Photoshop Elements 11 (panoramic stitching).

### Description approach

The description follows the concepts of describing the animal segment by segment, structure by structure (Haug, Briggs & Haug, 2012). The morphological terminology largely follows Beutel et al. (2013). Yet, to enhance the understandability for non-experts, we amended some of the special morphological terms with more general terms. As Insecta is an accepted ingroup of Crustacea s.l., more general eucrustacean and euarthropodan terms are additionally provided where necessary to provide wider frame correspondence. By providing both types of terms, experts as well as non-specialists can follow the description. This approach follows earlier attempts (Haug, Müller & Haug, 2018; Baranov et al., 2019).

### Registration of nomenclatural act

A single new species is described herein. The electronic version of this article will represent a published work according to the International Commission on Zoological Nomenclature (2012) (ICZN) and hence the new names contained in the electronic version are effectively published according to the ICZN from the electronic edition alone. This published work and the nomenclatural acts it contains have been registered in ZooBank, the online registration system of the ICZN. The ZooBank LSIDs (Life Science Identifiers) can be resolved and the associated information viewed through any standard web browser by appending the LSID to the prefix http://zoobank.org/. The LSID for this publication is urn:lsid:zoobank.org:pub:F85AEA78-527D-4B64-A48A-A5D21C425F8F. The online version of this work is archived and available from the following digital repositories: PeerJ, PubMed Central and CLOCKSS.

## Description

### General body organisation

**Figure 1:**
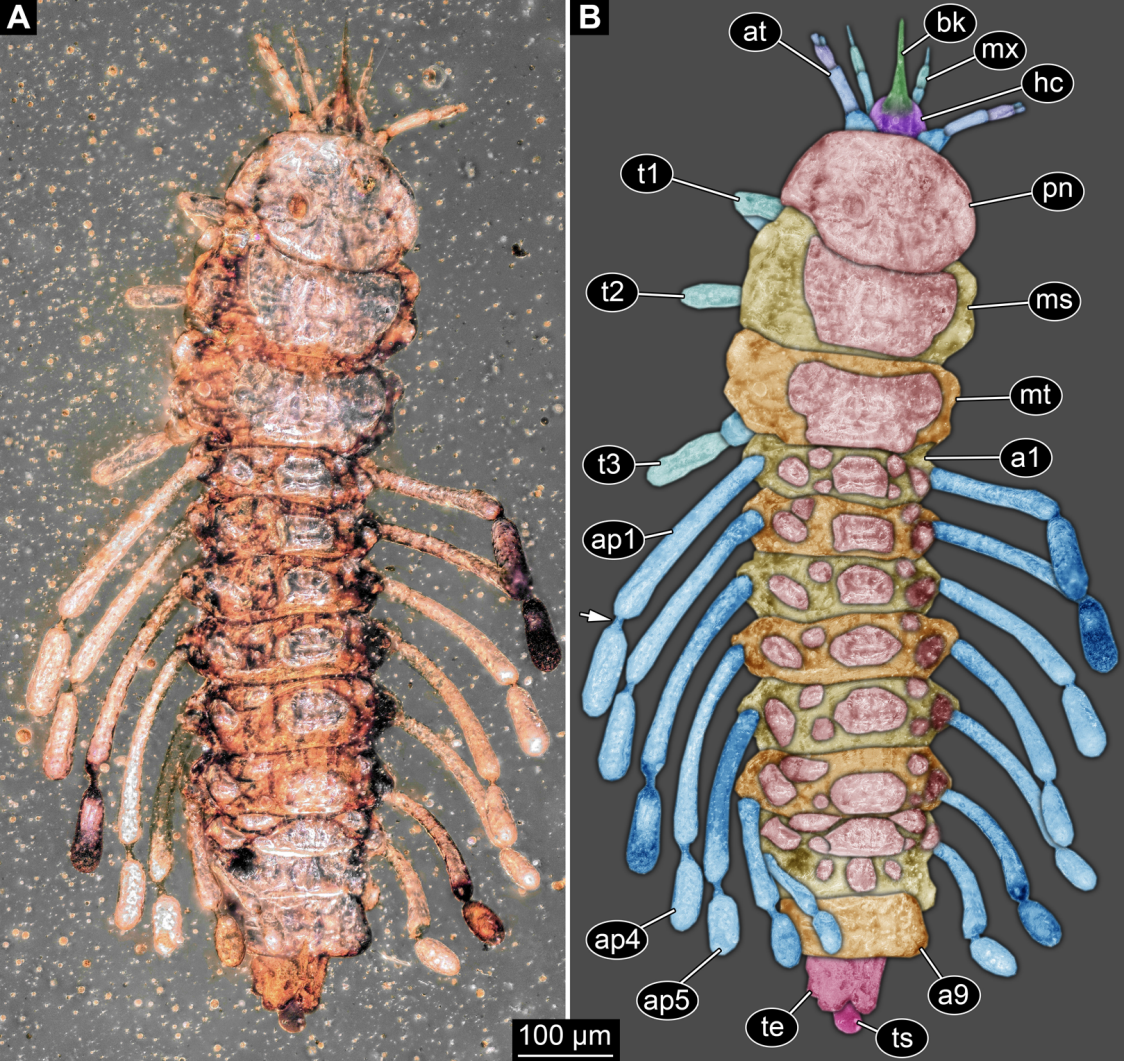
New fossil larva, dorsal view. (A) Composite image under ring-light illumination. (B) Interpretation presented as colour markings; arrow points to constriction. Abbreviations: a1–9, abdominal segments 1–9; ap1–5, abdominal processes 1–5; at, antenna; bk, beak, mouth cone; hc, head capsule; ms, mesothorax; mt, metathorax; mx, maxilla; pn, pronotum; t1–t3, thoracic appendages (‘legs’) 1–3; te, trunk end; ts, terminal structure.

**Figure 2:**
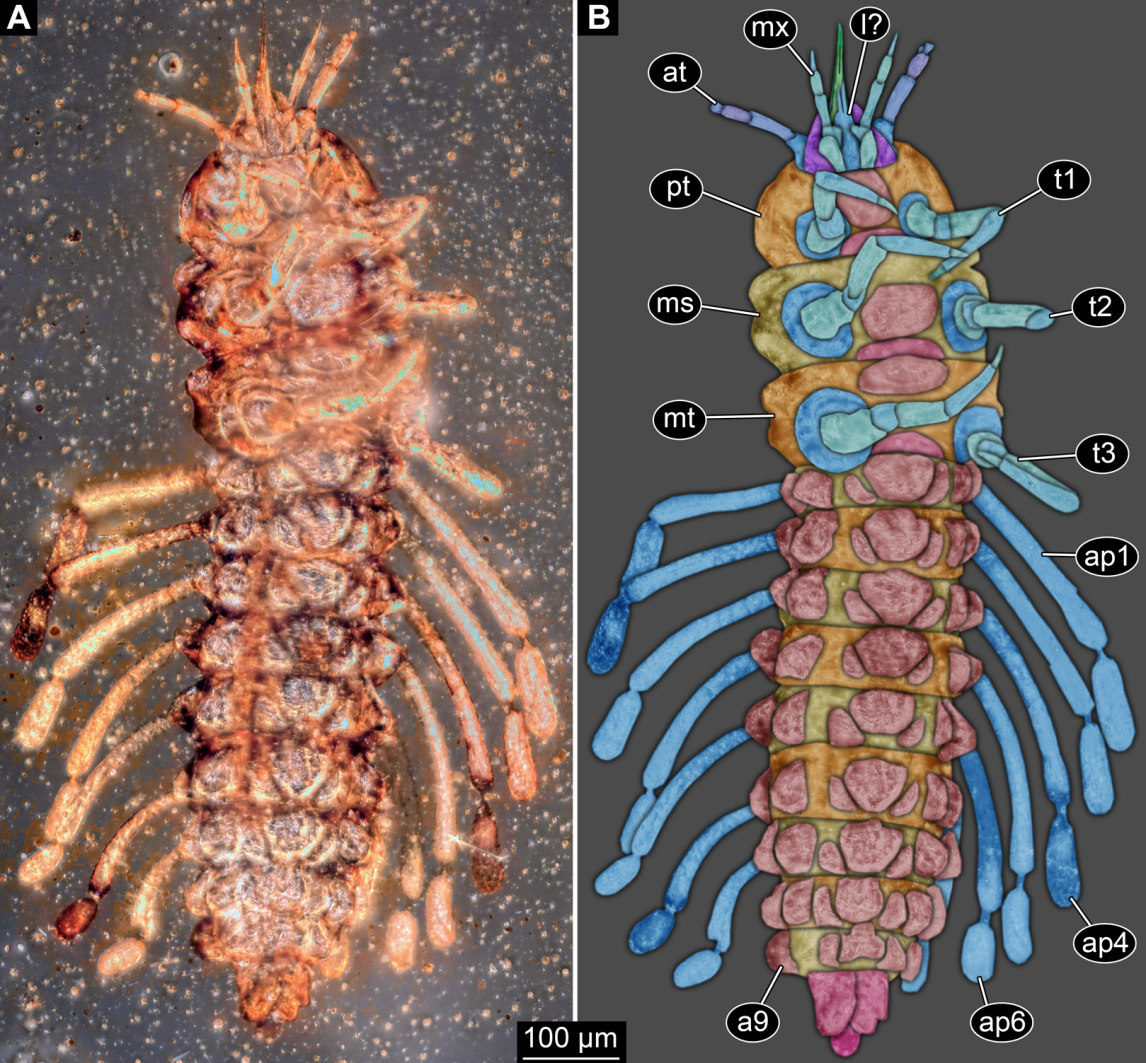
New fossil larva, ventral view. (A) Composite image under ring-light illumination. (B) Interpretation presented as colour markings. Abbreviations: a9, abdominal segment 9; ap1–6, abdominal processes 1–6; at, antenna; l?, possible labium; ms, mesothorax; mt, metathorax; mx, maxilla; pt, prothorax; t1–t3, thoracic appendages (‘legs’).

**Figure 3:**
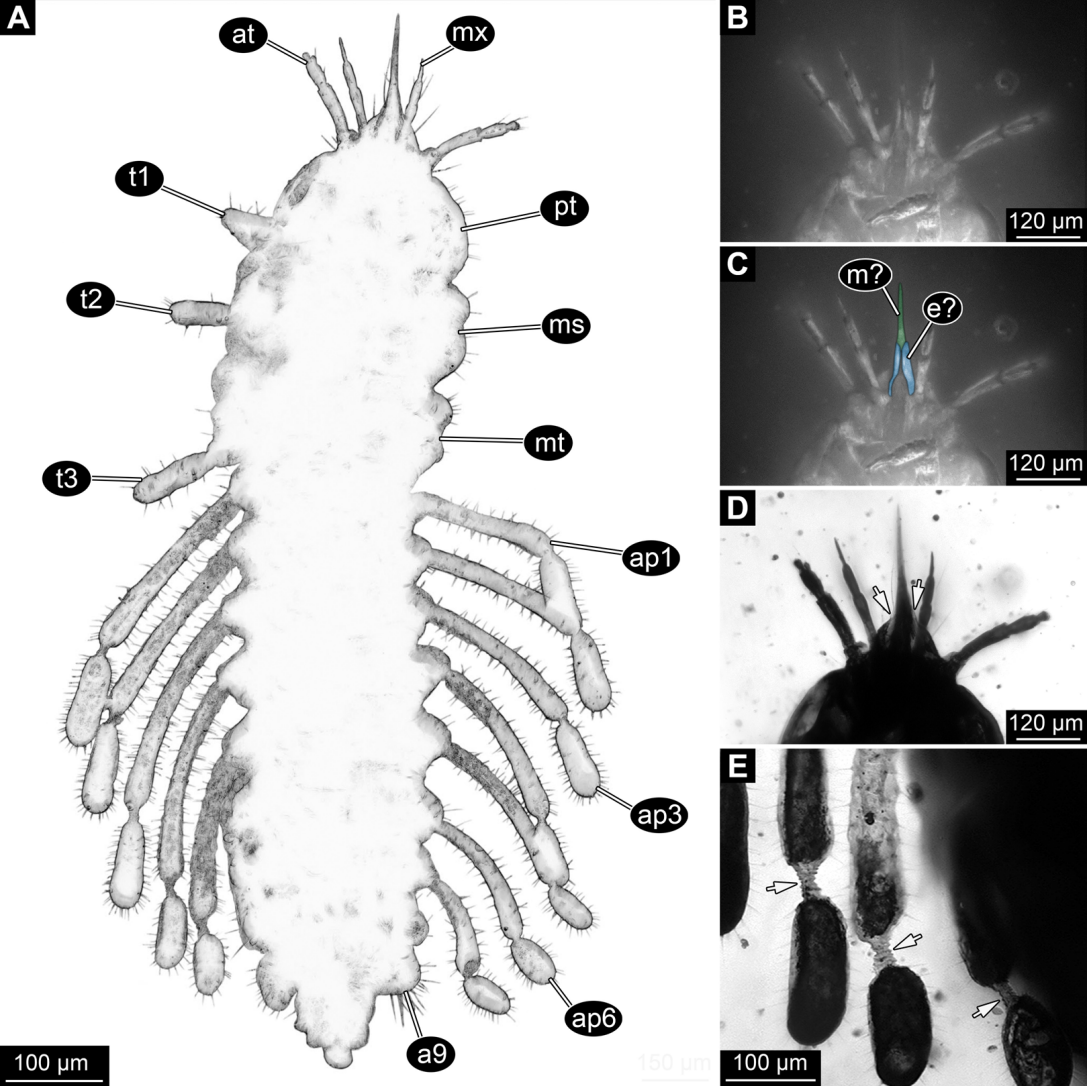
New fossil larva, ventral view. (A) Composite image under phase contrast image, processing following Haug et al. (2009b). (B) Composite image under fluorescence light; close-up of head region. (C) Interpretation of B presented as colour markings. (D and E) Composite image under transmitted light. (D) Close-up of head region; arrows mark light area, indicating the narrowness of the beak. (E) Close-up of abdominal processes arrow points to constrictions with wrinkles. Abbreviations: a9, abdominal segment 9; ap1–6, abdominal processes 1–6; at, antenna; ms, mesothorax; mt, metathorax; mx, maxilla; pt, prothorax; t1–t3, thoracic appendages (‘legs’).

Small larva with body organised in (presumably) 20 segments, ocular segment plus 19 post-ocular segments (Figs. 1, 2 and 3A). Ocular segments and post-ocular segments 1–5 forming head capsule, remaining segments represent the trunk (Figs. 1, 2 and 3A).

### Head region

Head capsule partly concealed by further posterior structures (Fig. 1). In dorsal view only the anterior (clypeal?) region of the head capsule is visible (Fig. 1). Dorsally visible part of the head capsule roughly triangular or trapezoidal in shape, with the narrower edge anterior. Ocular segment not externally discernible, that is, no eye structures apparent. It cannot be excluded that, comparable to larvae in some dipteran and hymenopteran lineages, the head capsule is partially retracted into the anterior trunk.

Appendages of post-ocular segment 1, antennae (antennulae in neutral euarthropodan terminology) identified by their dorsolateral insertion. Antenna subdivided into four elements (flagellomeres/antennomeres) (Fig. 2). Proximal element cone-shaped, tapering distally; proximal width almost half of the posterior edge of the head capsule, distal width only half as wide; about as long (proximal-distal dimension) as proximal width. Element 2 slightly narrower proximally; widening slightly distally; distal edge about as wide as distal edge of element 1. No sensillae visible. About two times as long as element 1. Element 3 smaller than preceding element; narrower proximally, widening further distally, but tapering again distally, resulting in a club-like shape. About 60% of the length of the preceding element. Element 4 even smaller. Slightly narrower proximally, widening very slightly. About as long as wide. Also arising from element 3, next to element 4, is a very small structure resembling element 4 in shape but being only about half as long and half as wide, probably a sensory sensilla.

Post-ocular segment 2 (intercalary segment) without recognisable structure (inferred presence).

Mouth parts (labrum, structure of ocular segment, plus post-ocular segments 3–5) forming a distinct elongated cone (‘beak’) with a narrow, pointed tip (apex). The dorsal side of the beak appears to be formed by a single homogeneous structure (Fig. 1), most likely representing the labrum. On the dorsal side and in the proximal half of the beak two pairs of long setae protrude antero-laterally (Fig. 3A).

On the ventral side of the cone several sub-structures are differentiable (Figs. 2, 3B–3D) most likely representing appendages, or parts of these, of post-ocular segments 3–5, that is, mandible, maxilla (maxillula in neutral euarthropodan terminology) and labium (maxilla in neutral euarthropodan terminology). Mandibles most likely represented by very faint straight lines of the beak.

Further posterior appendage, possible maxilla (yet unclear, also parts of labium cannot be excluded; Fig. 2) with five visible units (unclear if representing true jointed elements). Proximal unit (cardo?) elongate and prominent, subsequent unit (stipes?) also prominent but much shorter, only half as long as the preceding unit. Medially with distinct bulge. The further distal three units form a palp (endopod in neutral euarthropodan terminology) of the maxilla. Element 1 (‘palpomere’) of the maxillary palp distinctly narrower than preceding elements and elongate, about as long as element 2 of antenna, tapering towards the middle and widening again distally; element 2 shorter, with about 60% of the length of element 1; proximally slightly narrower than preceding element, somewhat widening towards the middle region, tapering again distally to about the width at the base of this element; element 3 distinctly spine-like, only slightly shorter than element 3 but distinctly more slender. The palps are of about the same length as the antennae and only slightly narrower. Longer bifurcated structure (Fig. 2) at about half of the length of the beak possibly representing thin elongated endites of the maxilla (galea or lacinia; alternative interpretation below).

Remaining visible structures of the mouth parts densely packed together, protruding anteriorly from between the maxillary palps, possibly representing the labium. Proximally conjoined appendage pair. Proximal part (possible mentum or postmentum) roughly rectangular element in ventral (posterior) view, constricted by the median bulges of the maxilla. Paired elongated structures with straight lateral margins and rounded median margins visible under fluorescence light on the ventral side in the proximal half of the beak (Figs. 3B and 3C), possibly representing proximal parts of palps of the labium, not visible in translucent light. Bifurcated structure at about half of the length of the beak could also represent the median enditic edge of the labium.

### Anterior trunk region, thorax

Post-ocular segments 6–8 sub-similar, forming a single functional unit (tagma), the thorax (Figs. 1 and 2). Thorax segment 1 dorsally distinctly sclerotised, forming a tergite (pronotum; Fig. 1). Pronotum twice as wide as the head capsule at widest point, partially concealing head and following segment. Anterior rim gently rounded, slightly narrower than posterior edge. Lateral edges gently curved; posterior edge also gently rounded, partially concealing further posterior structures. Ventrally (Fig. 2) with a distinct median sclerotisation (sternite). Sternite rounded, almost circular, occupying slightly less than 30% of the entire width, slightly shorter than entire segment. On each side of the sternite with a distinct walking appendage, inserting slightly more towards the posterior side of the segment. Most proximal element, coxa (most likely basipod in neutral euarthropodan terminology), almost as large as sternite, cone-shaped distinctly tapering distally. Further proximally surrounded by distinct ring-shaped fold (unclear if sclerotised, sub-coxa?). Due to the perspective, the exact length of coxa is unclear. Element 2, trochanter, short tube-shaped, slightly narrower than coxa at distal edge, about as long as broad (estimated, difficult due to perspective). Element 3, femur, at least twice the length of trochanter, similar diameter. Element 4, tibia, about as long as trochanter and femur combined, sightly more slender. Element 5, tarsus, is spine-like, tapering distally into sharp point, sightly curving inwards; slightly less than half of the length of the preceding element, distinctly more slender at base than the distal end of the preceding element. Ventral surface posterior to sternite, laterally surrounded by insertions of appendages, trapezoidal/trapezium-shaped membraneous area.

Thorax segment 2 in dorsal view (Fig. 1) slightly wider than preceding segment, about as long. Dorsally distinctly sclerotised, forming a tergite (mesonotum). Unlike the pronotum the sclerotised region does not occupy the entire dorsal region of the segment, but leaves the sides unsclerotised, membraneous. The sclerotised region is wider towards the anterior, about 60% of width, and narrows towards the posterior, about 40% of entire width. Ventrally sub-similar to preceding segment (Fig. 2), only differing in the length of the appendage elements which are slightly longer.

Thorax segment 3 sub-similar to preceding segment, about same dimensions. Also forming a dorsal sclerite (metanotum; Fig. 1), slightly shorter in anterior-posterior dimensions. Ventrally also sub-similar to anterior segments (Fig. 2). The only recognisable difference is the position of the median sclerite which is further anterior than in the preceding segment.

### Posterior trunk region, abdomen

Post-ocular segments 9–16 sub-similar, forming a single functional unit, the anterior abdomen (not corresponding to abdomen in other crustaceans). Abdomen segment 1 (post-ocular segment 9) slightly narrower than preceding thorax segment and significantly shorter (in anterior–posterior dimensions), about half as long. Set off from preceding segment by distinct fold (Figs. 1 and 2).

Dorsal region of the segment with 7 sclerites (Fig. 1). The central sclerite occupies less than 30% of the width, but only leaves a short unsclerotised region anteriorly and posteriorly. Sclerite roughly rectangular, but anterior corners truncated. Further sclerites arranged symmetrically on each side. Two pairs of sclerites right next to the central one, one further anterior, one further posterior. Both sub-square shaped, significantly smaller than central sclerite, anterior one slightly larger than posterior one. Further laterally one additional sclerite on each side, slightly larger than one of the pair of sclerites, rounded in shape. Ventral region also with 7 sclerites (Fig. 2). A central sclerite occupies less than 30% of the width, but only leaves a short unsclerotised region anteriorly and posteriorly. Sclerite symmetrical trapezoidal, longer edge facing forward. Sclerite flanked by a smaller rounded, crescent-shaped sclerite postero-laterally on each side. Further laterally, with some distance, a larger rounded sclerite on each side. Already partly on the functional lateral side of the body a further sclerite on each side. This latter sclerite appears to form a functional anchoring structure for a dorso-lateral projection or process, one on each side. The insertion areas of the lateral processes may represent true joints. Processes elongate, about 2x as long as the trunk part of the segment is wide. The diameter is comparable to that of the thorax appendages. Process curving gently posteriorly. At about three fifth of the entire length with a distinct constriction, resulting in a sausage-shaped distal part. The surface of the processes at the constriction has a wrinkled appearance distinct from other parts of the processes (Fig. 3E). Distal tip of this part gently rounded. Entire process armed with numerous short setae (Fig. 3A).

Abdomen segments 2–6 (post-ocular segment 10–14) sub-similar to abdomen segment 1. Sclerites slightly different in size, smaller ones not always very apparent. Processes progressively shorter towards the more posterior segments. Process of abdomen segment 6 about 60% of the length of that of abdomen segment 1.

Abdomen segments 7 and 8 (post-ocular segments 15–16) difficult to differentiate on the dorsal side, sub-similar to further anterior segment concerning size. Abdomen segment 7 with a sclerite arrangement comparable to that of the further anterior segments, yet central sclerite larger. Abdomen segment 8 with only a small central sclerite and a single further lateral sclerite on each side. Ventral region of both segments with a full set of sclerites (Fig. 2). In both segments the sclerites are arranged closer to each other compared to the further anterior segments. Abdomen segment 7 with a short process, abdomen segment 8 without a process.

Abdomen segment 9 (post-ocular segment 17) narrower, but longer dorsally than the preceding segments. Without apparent sclerites dorsally and ventrally, yet as a whole appearing slightly more sclerotised. Without process, yet postero-lateral corners slightly bulging, in this aspect resembling distal region of the abdomen processes in shape. Also, comparable to this region the corners carry numerous setae (Fig. 3A). Trunk end (possibly including abdomen segments 10 and 11, i.e. post-ocular segments 18 and 19) truncated cone-shaped. Narrower than preceding segment (about 50%) with a distinct sharp notch at the postero-dorsal edge, resulting in two lobe-like protrusions (Fig. 1). Similar arrangement ventrally (Fig. 2). Ventrally additionally with a distinct suture, dividing the trunk end into a left and right side (Fig. 2). Posteriorly arising is a short terminal structure, about the same diameter as the thorax appendages.

## Discussion

### Structural interpretation: mouth parts

The most astonishing feature of the fossil is the structural organisation of the mouth parts. Not all details are immediately accessible and therefore demand for a careful interpretation. Simply identified can be the proximal parts (cardo and/or stipes?) of the maxillae together with the distal palps. Furthermore, some of the remaining structures apparently form a single elongate structure, which can be loosely characterised as ‘mouth cone’, ‘beak’ or ‘stylet’. This structure is so tightly formed that it is difficult to clearly identify which mouth parts contribute here or which sub-parts of those are involved. Yet, it is pretty clear that either both mandibles and maxillae or at least one of them took part in the formation of the cone. It can not represent a single structure (also not other ones, such as the hypopharynx) due to the lines indicating that several structures are involved in the formation.

Especially under phase contrast illumination (Fig. 3A) a thin line is apparent in the middle of the cone. This either indicates a kind of seam between paired mouth part structures that make up the cone (in this case this would be most likely the mandibles), or the presence of further structures, for example, a sucking channel or delicate stylet-like mouth part within the cone. Additionally, a bi-forked structure is apparent ventrally, reaching two thirds of the entire length of the mouth cone (Fig. 2). The two ‘fork-teeth’ could represent endites, galeae or laciniae, of the maxillae or the distal end of a ligula (conjoined endites of the labium). In larvae of the beetle species *Glyptolopus quadricostatus* Heinze, 1944 (Ceryloninae) the mouth parts form a beak similar to that of the presented fossil and there is a bifurcate distal end representing the enditic edge of the labium (‘ligula’). However, in the case of *G. quadricostatus*, the ligula extends up to the apex of the mouth cone, yet in other species of Ceryloninae the labium is shorter.

Another paired structure of the mouth parts becomes apparent under fluorescent light (Figs. 3B and 3C). Proximal to the putative distal end of the labium (ligula) there are elongated elements that show a higher fluorescence capacity than the remaining part of the mouth cone. These structures could be interpreted as proximal elements of the labial palp. Judging from their position (‘grasping’ around the mouth cone) it can be hypothesised that they mechanically stabilise the mouth cone. A roughly comparable arrangement is known in the sucking mouth parts, factually a mouth cone, of heteropterans (true bugs; Wenk, Lucic & Betz, 2010).

It remains unclear to which extent the labrum is involved in forming the mouth cone. It could be possible that the labrum is short (and thus not discernible) and only the remaining mouth parts form the beak. However, it is also possible that the labrum completely conceals the remaining mouth parts in dorsal view and thus there is no margin of the labrum visible in the fossil (see “Discussion”). Despite the uncertainties it becomes clear that the mouth parts of the fossil indeed form a highly structured mouth cone, beak, pin or stylet. This is therefore interpreted as original structure and not an artefact caused by embedding into the resin.

### Systematic interpretation: general aspects

Although the specimen is in many aspects unusual on first sight, a rough systematic interpretation is easily possible. Its body is prominently subdivided into segments; a distinct head with jointed appendages is followed by an anterior trunk with three segments, each with a pair of prominent jointed appendages; these are followed by a series of posterior trunk segments that differ in morphology from the anterior segments. This principle body organisation immediately identifies the specimen as a representative of Insecta (= Hexapoda in Anglo-American literature).

Within Insecta, all non-pterygotan lineages can be excluded from further phylogenetic consideration, as the specimen has no indication of prominent processes on the posterior abdomen segments (cerci and/or terminal filament present in: Diplura: Sendra et al., 2006, 2016; Reboleira et al., 2010; Zygentoma: Espinasa et al., 2012; Smith, 2013; Archaeognatha: Sturm, 2009; Hädicke et al., 2014; Haug et al., 2015a; Zhang et al., 2018; furca as present in Collembola: Fjellberg, 1986; Hopkin, 1997; Hädicke, Haug & Haug, 2013) and mostly possesses a well developed antenna (absent in Protura; Böhm et al., 2011; Bai & Bu, 2013; Galli et al., 2016).

The absence of wings can thus be interpreted as caused by secondary absence or ontogenetic effects (not yet present). The combination of the following character states leads us to the interpretation of the fossil as a larval representative of Holometabola. The legs in the fossil larva are relatively short compared to the overall body size (long abdomen). Also the tarsus is not subdivided into multiple elements. The head is dorsally covered by the pronotum and the antennae are short and with uneven-sized elements. As the most characteristic feature we see the ‘incomplete’ sclerotisation of tergites and sternites of the abdomen segments (Figs. 1 and 2), resulting in multiple isolated sclerites per segment on the dorsal and the ventral side.

Many of these characteristics also fit to non-holometabolan groups of Insecta. Yet, the combination of all characters seems to be only present in larvae of Holometabola. Thus, we see the interpretation as a larval representative of Holometabola as well founded.

### Systematic interpretation: antennae

**Figure 4:**
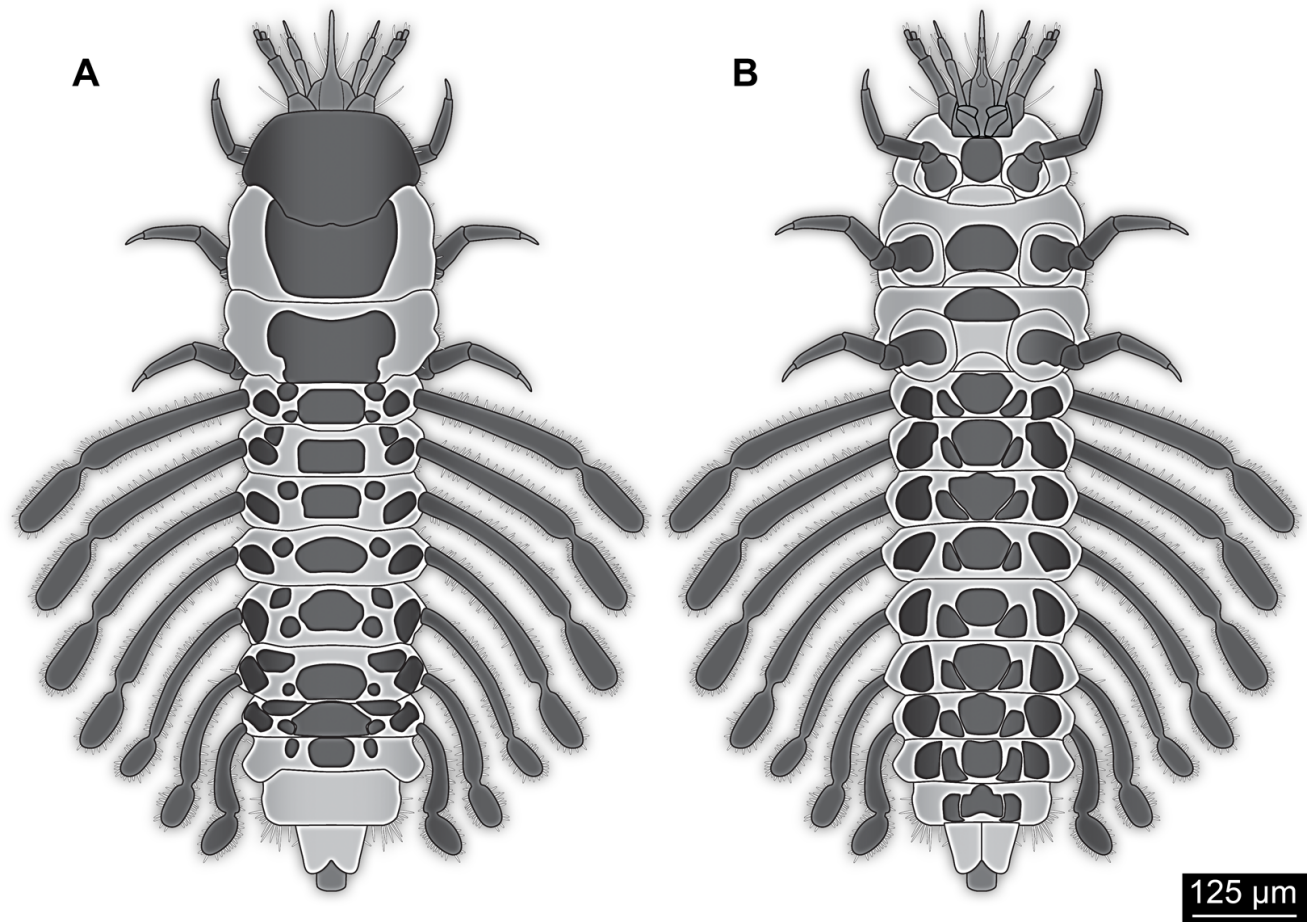
Simplified restoration of new fossil larva. (A) Dorsal view. (B) Ventral view.

The antenna in the fossil larva appears to consist of four elements (Figs. 1, 2, 3D and 4). On the distal end of the penultimate (third) element there is a prominent structure that resembles a sensorium known in many holometabolan larvae. The antenna is robust, but elongated with a long second element. In many larvae of Holometabola the antenna is short and inconspicuous, as for example in larvae of Hymenoptera (Wong, 1963; Heraty & Darling, 1984), Trichoptera (Wichard, Arens & Eisenbeis, 2002; Wichard, Gröhn & Seredszus, 2009) and Lepidoptera (Kristensen, 1984); in some it is rather bulbous as in larvae of Mecoptera (Jiang, Gao & Hua, 2015; Saltin, Haug & Haug, 2016); in others it is rather slender with elongated elements as in larvae of Neuroptera (Haug, Müller & Haug, 2019) and Raphidioptera (Aspöck, 2002; Beutel & Ge, 2008; Aspöck & Aspöck, 2009).

In Megaloptera the larvae also usually have four elements and a sensorium, yet, the sensorium is located on element 2 of the antenna. This has been suggested to be autapomorphic for the group (Beutel & Friedrich, 2008) and differs from the condition in the fossil larva.

In beetle larvae, when the antenna is not short, often the penultimate antenna element is elongated, similar to the condition in the fossil larva. Antennae with four elements, including a single flagellum element and a sensorium on the distal end of the penultimate element, occur in many lineages within Coleoptera. Yet, most larvae of Polyphaga possess antennae with three elements (Lawrence, 1991a; Lawrence & Slipinski, 2013). Also in some neuropteran larvae the antennae appear to bear a sensorium on the penultimate element; yet, they usually have more than four elements (Beutel, Friedrich & Aspöck, 2010). The antenna morphology of the fossil larva is hence well compatible with a relationship to certain coleopteran ingroups. It is worth noting, however, that the antennae of the fossil larva are much longer than those of most beetle larvae, including larvae of Ceryloninae, which normally have short antennae with only three elements (Lawrence & Slipinski, 2013).

### Systematic interpretation: mouth parts

One might argue that the mouth cone of the fossil is functionally prognathous, as are mouth parts in many larvae of Neuropterida, namely lacewings (Neuroptera; Beutel, Friedrich & Aspöck, 2010; Badano & Pantaleoni, 2014; Badano et al., 2017; Haug, Müller & Haug, 2019), snake flies (Raphidioptera; Aspöck, 2002; Beutel & Ge, 2008; Aspöck & Aspöck, 2009) and dobsonflies, fish flies and alderflies (Megaloptera; Beutel & Friedrich, 2008), as well as in many ingroups of Coleoptera (beetles; Krenn, 2007). Krenn (2007) had considered that the prognathous arrangement of mouth parts represents an autapomorphy of the larger group including Neuropterida and Coleoptera as ingroups (Neuropteriformia). However, Krenn (2007) also pointed out that there are cases of prognathy in larvae in other holometabolan groups as well. Hence, even if one would accept the mouth parts of the fossil as prognathous, there is little phylogenetic systematic signal in this notion.

**Figure 5:**
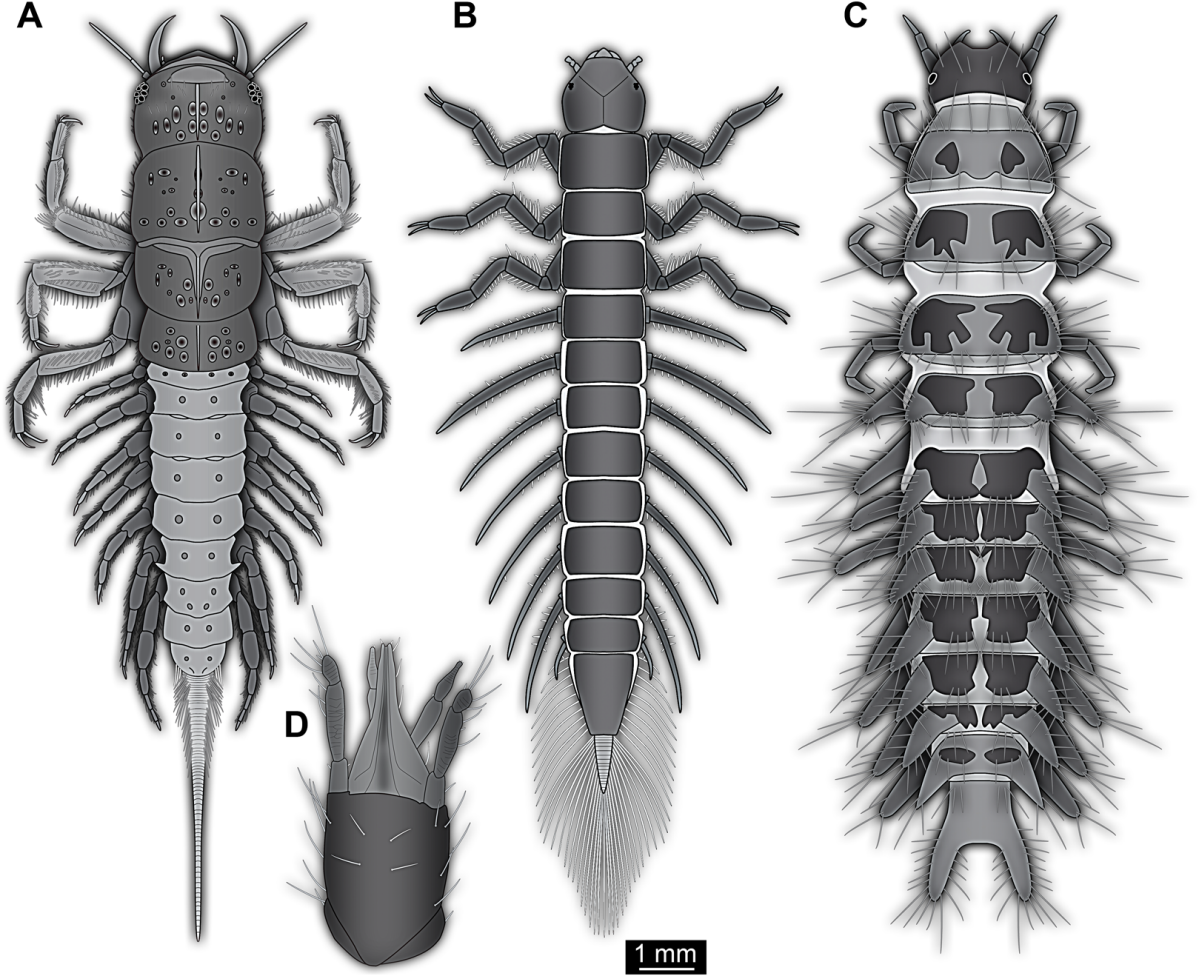
Larvae for comparison, redrawn from the literature. (A) Extant megalopteran larva (*Indosialis bannaensis*, Sialidae) (Bowles & Contreras-Ramos, 2016); total length 15.4 mm. (B) Fossil megalopteran larva (Ponomarenko, 2012). (C) Larva of the beetle group *Drilus* (Baalbergen, Schelfhorst & Schilthuizen, 2016); no scale available, but estimated length about 18 mm based on similar specimens. (D) Head of larval mantis lacewing (Dorey & Merritt, 2017); note how the piercing-stylets are positioned close to each other almost appearing as forming a single mouth cone; no scale available, but estimated length about 450 µm based on similar specimens.

Piercing-sucking mouth parts in larvae are rather unusual for holometabolans while cutting-grinding mouth parts are present in most groups. The probably most widely known piercing mouth parts in holometabolan larvae are those in Neuroptera. Here, mandible and maxilla (mainly one endite of it, possibly the galea) form a piercing stylet (Aspöck, Plant & Nemeschkal, 2001; Aspöck, Haring & Aspöck, 2012; Beutel, Friedrich & Aspöck, 2010). More precisely each mandible-maxilla complex is one piercing stylet, neuropteran larvae have hence two such stylets. These stylets can be curved or straight, depending on the subgroup of Neuroptera (cf. Haug, Müller & Haug, 2019, their fig. 4). In very few cases, such as in the larvae of mantis lacewings, a pair of straight stylets might appear as a single structure when both these stylets are held close together (Fig. 5D; Rice & Peck, 1991, their fig. 2; Hoffman & Brushwein, 1992; Dorey & Merritt, 2017, their fig. 4C). While in these cases the two stylets seem to form a single cone, this single ‘cone’ is much wider than the beak in the new larva. Still it cannot be entirely ruled out that the new larva has a comparable, but more slender arrangement. In this case palps would be labial palps. In this interpretation the head region could be understood as a ‘maxillary head’ as conceptualised by Aspöck & Aspöck (2007).

Therefore, affinities of the larva in focus of this study with Neuroptera cannot be ruled out. Although some structures are more difficult to understand, many Cretaceous lacewing larvae appear quite unusual (Badano et al., 2018).

Also some beetle larvae form such paired piercing devices. Piercing mouth parts are, for example, present in larvae of Dytiscidae. Larvae of this group have suction channels within the mandibles, similar to larvae of Neuroptera, but without the maxillae being involved in forming this channel (Balke, 2005). Also, beetle larvae of the group *Drilus* (Fig. 5C) possess piercing-type mandibles that are able to inject venom into their prey; still in these beetle larvae the mandibles appear quite normal, not being attached to any other mouth part or to each other and possessing a simple curved shape. The channel for injecting venom is apparently formed by a simple infold of the mandibular cuticle (Faucheux, 2014, his fig. 4c).

The mouth parts of the new specimen, forming a single stylet-like mouth cone, are even more unusual, at least for a larval representative of Holometabola. Yet, we could also consider that via a heterochronic shift adult characteristics have become expressed in a larval form, which seems in principle to be possible in holometabolans. For example, certain eye structures restricted to adults in most lineages can be shifted forward in ontogeny and already be present in the larvae (Melzer & Paulus, 1991).

**Figure 6:**
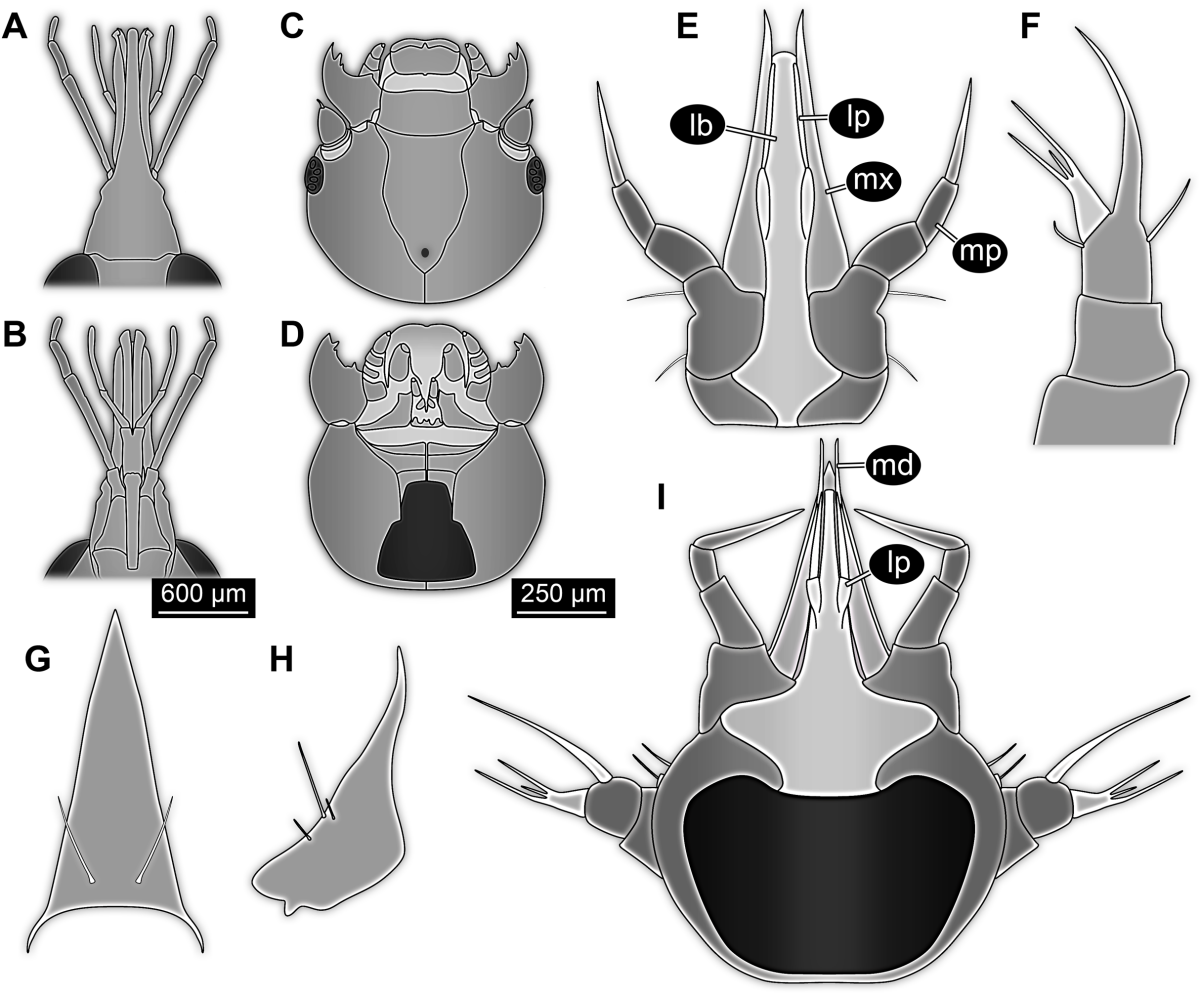
Details of heads and mouth parts for comparison. (A–D) Mouth parts of mecopterans (*Bittacus*). (A) and (B) Adult (Ma, Huang & Hua, 2014). (A) Dorsal view. (B) Ventral view. (C) and (D) Larva (first instar) (Tan & Hua, 2009, figs. 8 and 10). (E–I) Larvae of the beetle group Ceryloninae. (E–G) *Mychocerus hintoni* (Ślipinski, 1991). (E) Head, posterior view. (F) Antenna, sensorium in light grey. (G) Labrum. (H) Mandible of larva of *Mymicholeva acutifrons*, posterior view (Newton, 1991). (I) Head of *Philothermus glabriculus* (Ślipinski, 1991), posterior view. No scales available for (E–I).

Within Mecoptera some adult representatives have a distinct beak-like mouth part morphology (Ma, Huang & Hua, 2014; Saltin, Haug & Haug, 2016); the labrum is conjoined with the clypeus and forms a pointed tip that dorsally covers the distinctly elongated mandibles and laciniae of the maxillae (Figs. 6A and 6B). However, neither mandibles nor maxillae are extremely narrow and stylet-like. The mecopteran larvae, in contrast, have ‘regular’ cutting-grinding mouth parts (Tan & Hua, 2009; Saltin, Haug & Haug, 2016; Figs. 6C and 6D). Adults of some early representatives of the mecopteran lineage, however, such as *Lichnomesopsyche gloriae*, possessed long, piercing mouthparts, probably specialised for gymnosperm pollination (Ren, Labandeira & Shih, 2010).

Larvae of some species of Rypobiini (ingroup of Cucujoidea, more precisely Corylophinae, the group of minute fungus beetles) are quite extreme regarding their mouth part morphology. In species of the group *Holopsis* (ingroup of Rypobiini), the larvae have an enormously elongated anterior head, yet with cutting-grinding mouth parts anchored at the tip of this pseudo-beak, comparable to the condition in adult weevils (Curculionoidea; Yavorskaya et al., 2014).

In Eucinetidae (known as plate-thigh beetles; ingroup of Scirtoidea) the modifications seem to be more drastic. Not only the apical angle of the labrum is more pointed, also the endites (‘mala’, galea and/or lacinia of the maxilla) are more elongated and pointed than in representatives of Rypobiini. Similar to representatives of Rypobiini, the mouth parts of the larvae are of the cutting-grinding type (Vit, 1977, 1981; Leschen, 2005). While stylet-like mouth cones are present in adults of some of the major ingroups of Holometabola (e.g. some dipteran ectoparasites, such as representatives of Culicomorpha), a morphology comparable to that of the here presented larva is only present in larvae of two coleopteran groups.

In *Myrmicholeva* (ingroup of Staphylinoidea, Leiodidae, and an ingroup of Camiarinae) larvae and adults have been reported to possess piercing-sucking mouth parts arranged to an unpaired beak-like stylet (Newton, 1998, 2005). However, judging from the only illustration (Newton, 1991; Fig. 6H) available for larvae of *Myrmicholeva—*a drawing of an isolated mandible—the modification of the mouth parts is not as pronounced beak-like as, for example, in adult forms of Eucinetidae and also not as extreme as in the larva presented herein.

In species of Ceryloninae (ingroup of Coccinelloidea and Cerylonidae, the group of minute bark beetles) the arrangement of the mouth parts in adults and larvae results in a very slender beak, quite comparable to the condition in the fossil larva (Figs. 6E, 6G and 6I). The labium is ‘constricted’ by the cardi of the adjoining maxillae (Costa, Ide & Teixeira, 1995). This appears to be the case also in the fossil larva (Fig. 2). The mouth cone of larvae of Ceryloninae tightly incorporates the labrum dorsally, and only the tips of the mandibles protrude beyond the labrum (Costa, Ide & Teixeira, 1995; Fig. 6I). Also, the labrum and the clypeal region of the head capsule are conjoined (Ślipinśki & Lawrence, 2010). Such a morphology also leads to a homogeneous appearance of the mouth cone in dorsal view, similar to the condition in the fossil larva.

While there are so many similarities of the fossil larvae to those of Ceryloninae in mouth part morphology, there is one crucial difference: In the fossil the mouth parts are distinctly oriented forward, while in larvae of Ceryloninae the mouth parts are directed downwards to backwards.

In summary, slender beak-like mouth parts are quite rare among holometabolan larvae, but the structure of the mouth parts of the larvae within the focus of the study seems to be compatible with morphologies most often occurring in Neuropteriformia.

### Systematic interpretation: habitus

**Figure 7:**
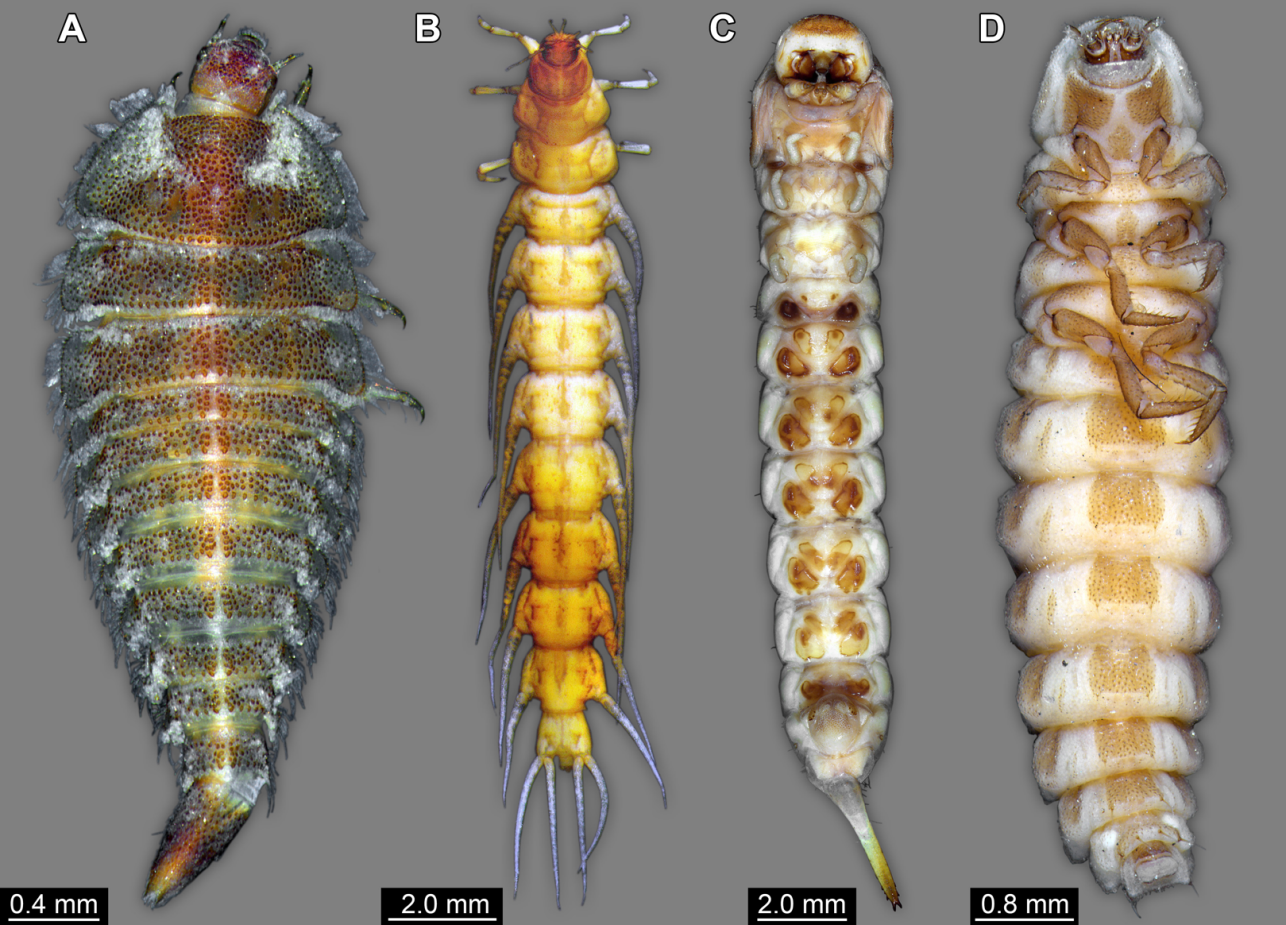
Coleopteran larvae for comparison, composite images. (A) *Helmis maugei* (Elmidae; ZMH 62854). (B) *Gyrinus* sp. (Gyrinidae; ZMH 62827). (C) *Hylecoetus dermestoides* (Lymexlionidae; ZMH 62844). (D) *Lampyris* sp. (Lampyridae; ZMH 62686).

**Figure 8:**
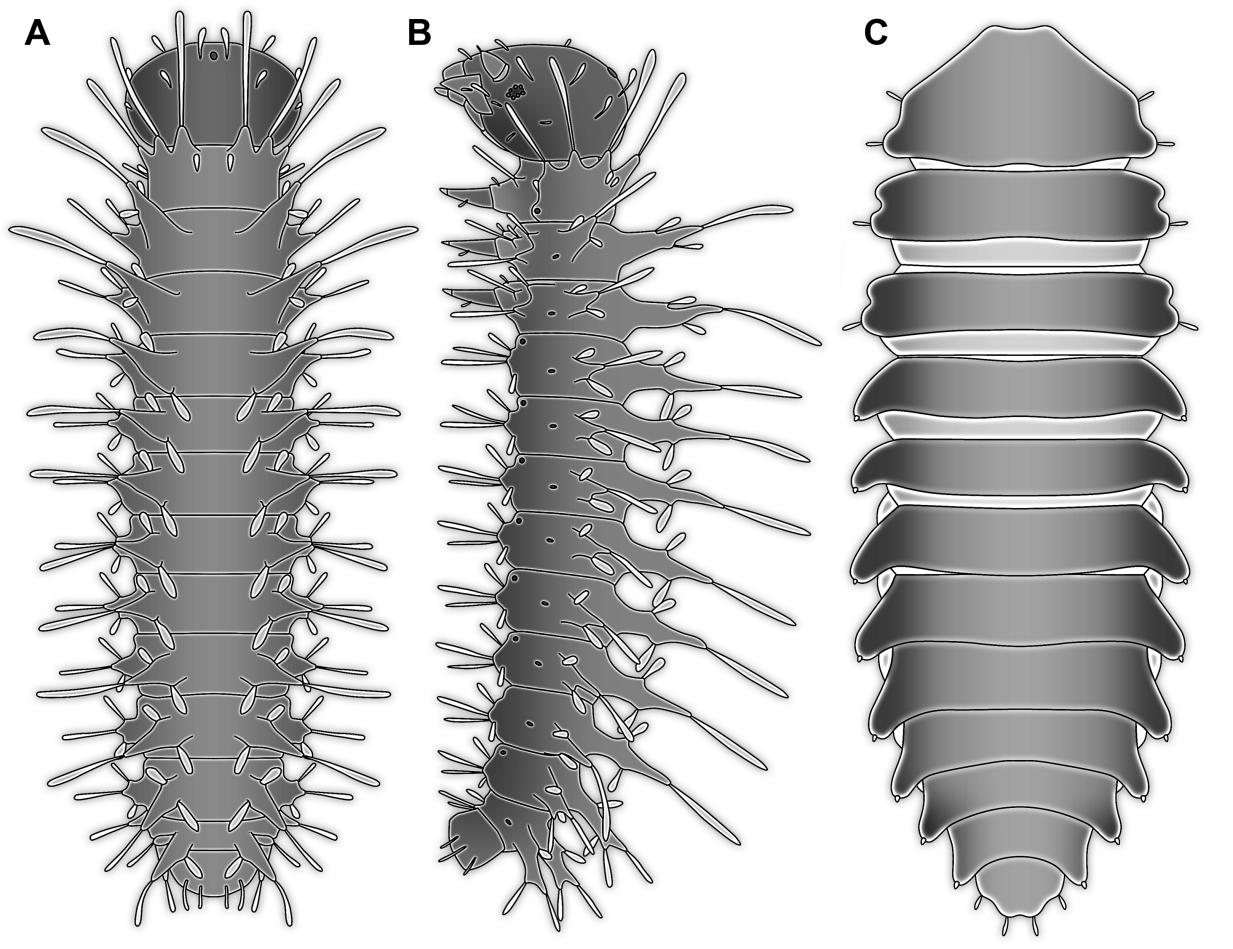
Larvae for comparison, redrawn from the literature. (A) and (B) Mecopteran larva (*Bittacus*; Setty, 1940); no scales available. (A) Dorsal view. (B) Lateral view. (C) Beetle larva (*Mychocercus* sp.; Lawrence, 1991b).

It is apparent that the lateral margins of the dorsal side are drawn out laterally and the prothoracic tergite (pronotum) anteriorly. This is quite unusual for most holometabolan larvae, but is found in several lineages, for example, among beetle larvae (Figs. 7A and 8C).

The strong subdivision of tergites and sternites into numerous distinct sclerites is also quite unusual. To a certain degree, sub-divided tergites are known in larvae of representatives of Sisyridae, Nevrorthidae, Osmylidae and Berothidae (all Neuroptera; Gepp, 1984; Aspöck & Aspöck, 2007). Yet, also in different beetle larvae (Figs. 7C and 7D) quite differently sclerotised regions are apparent, for example, in larval forms of the group *Drilus* (Elateridae; Baalbergen, Schelfhorst & Schilthuizen, 2016) as well as Chrysomelidae (i.e. *Phytodecta viminalis*; Dettner & Schwinger, 1987). Similar subdivisions between sclerites are also present in some mecopteran larvae (e.g. Panorpidae; Chen & Hua, 2011). Hence, also this aspect of the morphology does not provide a strong indication for a relationship of the fossil to a distinct ingroup, yet would be well compatible with a general position within Neuropteriformia.

### Systematic interpretation: abdomen processes

The abdomen processes dominate the overall appearance of the fossil larva. On a first glimpse, they are distantly reminiscent of the abdomen gills of mayfly larvae, but in fact even more so of the abdomen gills of megalopteran larvae (Fig. 5A).

In caterpillars (Lepidoptera), but also in the similarly eruciform larvae of Mecoptera (Tan & Hua, 2008, 2009; Jiang, Gao & Hua, 2015) and larvae of quite some lineages of Coleoptera (e.g. Coccinellidae, Chrysomelidae, Elateridae, Lycidae or Hydrophilidae; Lawrence, 1991a; Lawrence et al., 2011; Minoshima & Hayashi, 2015) there are also prominent protrusions arising from the abdomen. However, these projections differ in the morphology of the proximal region of the processes from the fossil larva. In many of these larvae the proximal region of the processes is very broad, and the protrusions gradually taper distally. This is even true for elongated slender processes as seen in some aquatic beetle larvae (cf. Fig. 7B).

The dorso-lateral and lateral processes in larvae of Bittacidae (Mecoptera) superficially resemble the processes in the here described fossil larva in a way that they appear subdivided (Figs. 8A and 8B). Yet, this seeming ‘subdivision’ in mecopteran larvae arises from the special morphology of the processes: from the proximal part a club-shaped seta arises distally (Tan & Hua, 2008, 2009; Jiang, Gao & Hua, 2015) which can cause the impression of a constriction, similar to the one seen in the processes of the fossil larva.

Within Megaloptera, larvae possess jointed lateral processes on the abdomen that are functional gills (Figs. 5A and 5B). In larvae of the megalopteran ingroup Corydalidae, these processes are elongate and tapering distally and are not subdivided into several elements (Gepp, 1984). In larvae of the megalopteran ingroup Sialidae these processes also taper distally, but are further subdivided into multiple elements (New & Theischinger, 1993; Bowles & Contreras-Ramos, 2016). The abdomen processes in Megaloptera can bear numerous setae, very similar to the condition in the fossil larva. However, the processes of megalopteran larvae also distinctly differ from those in the fossil. The processes in the fossil larva do not taper towards the tip, but end in a balloon-like distal region that is set off from the proximal region of the process in a very specific way. Unlike the subdivision into multiple articles as seen in megalopteran larvae, the distal region in the fossil larva is separated by a distinct constriction. This constriction has a conspicuous wrinkled surface structure (Fig. 3E).

Only more distantly comparable to the structure in the fossil are the gills of larvae of spongilla flies (Sisyridae, ingroup of Neuroptera). They are much smaller and do not protrude laterally from the body, but are only apparent in ventral view (Aspöck & Aspöck, 2007).

Wrinkled surfaces comparable to those in the here described fossil can be seen in spiracular gills of aquatic beetle larvae, such as those of Torridincolidae (Mixophaga; Vanin & Costa, 2001). The abdomen processes in larvae of Torridincolidae can also be further subdivided into distinct elements (Hájek & Fikáček, 2008). Yet, it needs to be pointed out that the wrinkled surface in larvae of Torridincolidae is located in the very proximal region of the process, at the joint between the process and the body proper and not further distally subdividing the process.

Larval lacewings are also known to possess distinct processes on their backs. These appear to be more prominent in larvae also known from Cretaceous ambers (Pérez-De la Fuente et al., 2012, 2016, 2018, 2019; Wang et al., 2016; Badano et al., 2018). Yet, these processes differ strongly from the ones in the new fossil larva. In lacewing larvae they arise further dorsally, are not jointed, often much more elongate and slender and show numerous branchings. These processes seem to be involved in carrying the camouflaging cloak (Wang et al., 2016; Pérez-De la Fuente et al., 2018). The orientation of the processes in the new larva appears not suitable for such a use, also there are no traces of any debris directly associated with the processes.

Also larvae of some extinct groups of Holometabola possess lateral processes on the abdomen. However, due to the relatively poor preservation of the fossils, the fine structure of the processes remains unclear (Rasnitsyn & Quicke, 2002; Novokshonov & Zhuzhgova, 2004). Among the most striking examples of such now extinct holometabolan larvae are those of the enigmatic group Miomoptera, more precisely larvae interpreted as representatives of *Permosialis*. These possess paired lateral processes at the posterior trunk units 1–9 (Sharov, 1953). It is not entirely clear whether these processes were subdivided into elements, but from the available images this does not seem to be the case (Sharov, 1953; Rasnitsyn & Quicke, 2002). Finally, also the Carboniferous holometabolan larva *Srokalarva berthei* possessed abdomen processes that were subdivided, reminding of those of megalopteran larvae (Haug et al., 2015b).

In summary, we can state that there are numerous examples among holometabolan larvae that possess abdomen processes. Yet, in detail they all differ from the morphology seen in the fossil larva. Therefore, also this character does not provide a strong phylogenetic signal.

### Systematic interpretation: the terminal end

So far, many characters of the fossil seem either best compatible with a closer relationship of certain ingroups of Coleoptera, Megaloptera or Neuroptera. The terminal end of the fossil is therefore compared to the corresponding body part of representatives of these groups. Within Megaloptera, larvae of Chaulididae and Corydalidae have paired terminal structures equipped with hooks (Gepp, 1984); only larvae of Sialidae have an unpaired structure as in the fossil larva. Yet, in some larvae of Sialidae this unpaired structure is strongly elongated (unfortunately termed ‘terminal filament’) and tapering distally (Fig. 5A; Bowles & Contreras-Ramos, 2016). In fossil representatives of the lineage it is less elongated, but also tapering distally (Fig. 5B; Ponomarenko, 2012). One could hence argue that the structure in the fossil is an equivalent of the structure in larvae of Sialidae, being even less elongated; yet, also here the differences in details do not provide a strong case. The unpaired terminal structure could also represent a not expanded pygopod (modification of abdomen segment 10) like it is present in many groups of holometabolan insects, including larvae of Coleoptera and some larvae of Neuroptera.

Coleopteran larvae seem either to possess paired terminal structures on abdomen segment 9 (‘urogomphi’) as, for example, in Leiodidae (Newton, 2005) and many other groups, or rather undifferentiated simple terminal ends, such as those in Ceryloninae. The trunk end of the larva is therefore again quite unique. It does not provide us with a strong phylogenetic signal.

### Systematic interpretation: summary

The new fossil larva shows an unusual combination of characters; some of these characters can be seen in different lineages of Holometabola. Still, as a more general observation only in larvae of Neuroptera and Coleoptera all of these characters are realised (piercing mouth parts, isolated sclerites on the dorsal and ventral side of the abdomen, long lateral abdomen processes). Yet, there is no extant coleopteran or neuropteran larva combining all characters we see in the fossil larva. The observed characters in the fossil larvae are well compatible with a position within Neuropteriformia, with no further elaboration currently possible.

Piercing mouth parts occur in multiple lineages of Coleoptera (Myrmicholeva, Eucinetidae, Rypobiini & Cerylonidae), but mostly in adults (Lawrence & Stephan, 1975; Lawrence, 1991b; Newton, 1991; Leschen, 2005; Ślipiński, Tomaszewska & Lawrence, 2009). The degree of modification of the mouth parts to form a very narrow stylet-like beak as in the fossil larva as well as the presence of piercing mouth parts in the larvae is only realised in the group Ceryloninae. However, it seems also possible that larval piercing mouth parts could have evolved in other coleopteran groups as well. Heterochronic changes in the ontogenetic development could, for example, lead to larvae with piercing mouth parts in groups where such a morphology is already present in the adults. While such heterochronic shifts seem not to be considered very often in holometabolan lineages, there are quite some prominent examples for such cases (e.g. presence of compound eyes in larvae; Melzer & Paulus, 1991).

No clear apomorphic characters of Coleoptera are apparent in the fossil, yet here we face the general challenge that many such characters are either based on adults (such as characters of the wings) or, if based on larvae, are not easily accessible in a fossil (such as aspects of muscles). Thus, it is also possible that the fossil is not part of any larger modern ingroup of Holometabola, but is a representative of a different branch of Holometabola that developed piercing mouth parts independently.

The ‘soft’ similarities with larvae of Coleoptera, Megaloptera and Neuroptera provide a certain possibility that the new fossil larva is a representative of the larger group comprising these three, Neuropteriformia. A more narrow interpretation is, as lain out above, not possible in a conflict free way.

### Taxonomy

Insecta (sensu Hennig, 1953; = Hexapoda Blainville, 1816)

Holometabola Burmeister, 1835 (= Endopterygota Sharp, 1898)

Neuropteriformia Ax, 2000

*Partisaniferus* gen. nov.

Life Science Identifier: urn:lsid:zoobank.org:act:AC782D9D-01B1-4372-A301-E1378D341F23

Etymology: Named in reference to the shape of the blade of some mediaeval weapons, the partisan and the resemblance of the shape of the beak of the larva; ‘-ferus’ for ‘carrying’.

Type species: *Partisaniferus atrickmuelleri* sp. nov.

Life Science Identifier: urn:lsid:zoobank.org:act:00196689-3460-4326-8AED-BB04AA34559F

Remark: As the genus is monotypic we cannot differentiate which characters are diagnostic for the genus or for the species. The genus is erected following the requirements of the ICZN. Alternative procedures are available and have been applied (Lanham, 1965; Béthoux, 2007a, 2007b, 2010; Béthoux et al., 2012; Haug & Haug, 2016) but currently lack broader support.

*Partisaniferus atrickmuelleri* sp. nov.

Etymology: after Patrick Müller, a German collector and hobby palaeontologist strongly supporting the field of amber research, who generously provided the holotype for scientific examination. In normal text, when added to the abbreviated genus name it will read ‘*P. atrickmuelleri’*.

Holotype: BUB 2787, former collection of Patrick Müller (Käshofen, Germany), now deposited in the Staatliche Naturwissenschaftliche Sammlungen Bayerns–Bayerische Staatssammlung für Paläontologie und Geologie in Munich under SNSB–BSPG 2019 I 171.

Ontogenetic stage of the type: larva of unknown instar number.

Type location: near Nojie Bum, Hukawng Valley, Kachin State, Myanmar (Burma).

Type stratum and corresponding age: unknown stratum, 98.8 million years, lowermost Cenomanian, lowermost Upper Cretaceous, after Shi et al. (2012), stratigraphically supported by a recent record of an ammonite shell of *Puzosia* (*Bhimaites*) in Burmese amber (Yu et al., 2019).

Differential diagnosis: Larva of a holometabolan. Mouth parts forming forward oriented piercing-sucking beak. Elongated lateral processes on abdomen segments 1–7, these processes with distinct constrictions in the distal part; dorsal and ventral side of abdomen segments 1–7 covered with up to seven isolated sclerotised plates each. Terminal end with a short, unpaired structure.

### Possible life habits of the fossil larva

The stylet-like mouth parts indicate that the larva was a piercing-sucking feeder. This does yet not immediately tell whether the larva was raptorial or sucking on plants or fungi. Therefore, given the absence of surrounding food sources, it must remain unclear which type of food the larva might have consumed. At this point it however needs to be highlighted that all beetle lineages with piercing sucking mouth parts similar to those in the fossil are somehow associated with fungivory (Lawrence, 1991b; Leschen, 2005; Newton, 2005; Ślipinśki & Lawrence, 2010; Ślipinśki, Lawrence & Cline, 2010). Thus, in combination with a small body size (that all of these beetle groups share), fungivory could be a driving force for the evolution of piercing mouth parts.

Some larvae of Ceryloninae, which have certain morphological similarities with this larva, live under bark (Lawrence, 1991b), and some other larvae of Cerylonidae even inhabit tunnels of wood-boring insects (Schedl, 1962). The exact food source of those wood-related species is still unknown, but it is likely that they also feed on fungi (Ślipinśki & Lawrence, 2010). A lifestyle similar to this provides a plausible interpretation for the embedment of the larvae in resin. However, in this interpretation the presence of long processes on the abdomen is odd, if not entirely inappropriate.

Even the general environment must remain unclear. While the prominent abdomen processes in the fossil resemble structures that function as gills in other larvae, which are aquatic, also terrestrial larvae possess comparable structures (e.g. representatives of *Drilus*). It is more unlikely, though not impossible, to see aquatic animals from such habitats to be trapped in amber (Wichard, Gröhn & Seredszus, 2009). In fact, aquatic larvae of Megaloptera (Sialidae) and Neuroptera (Nevrorthidae; Sisyridae) have been found preserved in amber (Wichard, Gröhn & Seredszus, 2009). In recent years, numerous animals which would be considered unlikely to be preserved in fossiliferous resins, such as non-avian forms of Dinosauria and birds (Xing et al., 2016) and an ammonite shell (Yu et al., 2019) have been found. In summary, we cannot really narrow down how the fossil larva might have lived, though aquatic habitats do present an attractive possibility. As the fossil larva appears to be that of a holometabolan species, speculations on the adult lifestyle would not be meaningful, as the morphology and the ecology can change tremendously in holometabolans during the metamorphosis.

### Diversity of the Cretaceous fauna

The Cretaceous fauna seems to be especially diverse due to three factors (Haug, Müller & Haug, 2019): (1) The survival of ‘old’ morphologies known from the Palaeozoic, but extinct after the Cretaceous, as exemplified by certain mayflies (Godunko, Staniczek & Bechly, 2011) or cockroach-like dictyopterans (Hörnig et al., 2018). (2) The emergence and diversification of new and distinctive morphological and functional types, such as ants and bees (Dlussky, 1996; Anderson, 2009). (3) The presence of early offshoots of diversifying lineages with ‘experimental’ morphologies that went extinct shortly thereafter. Examples of this latter case include representatives of the ‘vertical trap-jaw’ ants (Barden & Grimaldi, 2012; McKellar, Glasier & Engel, 2013) or the bizarre representatives of Dictyoptera, Alienoptera (Bai et al., 2016, 2018; Wipfler et al., 2019).

Besides these aberrant appearing adult forms also larvae are known in the Cretaceous that lack counterparts in the modern fauna (Badano et al., 2018; Haug, Müller & Haug, 2019). The new larva adds to this category, expanding the diversity of larval forms. This is especially astonishing as the new larva seems possibly a representative of Neuropteriformia, which is often considered to be the most diverse modern group, in terms of number of described species, mostly due to the ingroup Coleoptera (logical error of such statements outlined in Haug, Haug & Garwood (2016)). Hence, even in this group that is so rich and diverse in forms and species in the modern fauna, we find forms in the past that expand the overall ‘possibilities’ of the morphology and hence its morphological diversity. Also, it expands the overall diversity of the Cretaceous fauna. With this, the expansion of the morphological diversity very likely also represents an expansion of the ecological diversity. Comparable mouth parts in a larva are so far unknown in the same deposit, therefore the ecological function of the new larva has been so far most likely not reported for the Cretaceous fauna.

We cannot entirely exclude the possibility that we in fact have comparable forms in the modern fauna. So far, only very few species, for example, of Ceryloninae (and many closely related lineages) are known concerning their larvae. It might therefore be well possible that we could still discover a similar morphology in the modern fauna in the future.

### Convergence as a major factor for form diversity

The new larva is very unusual in its overall appearance. Yet, the aspect of being special is mostly related to the very special combination of characters. We know piercing mouth parts forming a narrow beak in larvae of several lineages of Insecta and Holometabola. Prognathous orientation of the mouth parts occurs also in numerous lineages. Similarly, the numerous sclerites per segment are known in different lineages, as are the abdomen processes, that is, in Sialidae, Sisyridae etc. (Beutel et al., 2013). Even the very unusually appearing joint between the proximal and distal part of the process seems to occur in other groups (e.g. Sialidae). It somehow appears as if there is a certain set of characters that can be combined in almost every possible way. A similar case within Neuroptera was recently reported by Haug et al. (2019).

Convergent evolution of characters seems therefore quite common within Holometabola. The concept what ‘convergent evolution’ means is still very underdeveloped. It refers to several independent underlying processes that lead to similar appearing morphologies. Yet, these may represent: (1) true independent novelties; (2) characters that were present in early representatives of the lineage, then lost in later forms, but then ‘re-activated’ in deeply nested ingroups; (3) repetitive expression of characters that seem to be present as potential from a genetic basis already in early ancestors (Hu et al., 2018).

Convergent evolution of specific structures can lead to new combinations of characters. The new larva is a further, quite extreme example of such a case by possessing a very distinct, unexpected type of morphology.

## Additional Information and Declarations

### Data Availability

The following information was supplied regarding data availability: The holotype is deposited in the Staatliche Naturwissenschaftliche Sammlungen Bayerns—Bayerische Staatssammlung für Paläontologie und Geologie in Munich under SNSB—BSPG 2019 I 171. Comparative extant material are available at the entomological collection of the Centrum für Naturkunde (CeNak), Hamburg. Specimens are registered under the repository numbers ZMH 62827, 62844, 62854 and 62686.

### New Species Registration

The following information was supplied regarding the registration of a newly described species: Publication LSID: urn:lsid:zoobank.org:pub:F85AEA78-527D-4B64-A48A-A5D21C425F8F. Partisaniferus LSID: urn:lsid:zoobank.org:act:AC782D9D-01B1-4372-A301-E1378D341F23. Partisaniferus atrickmuelleri LSID: urn:lsid:zoobank.org:act:00196689-3460-4326-8AED-BB04AA34559F.
